# Asia-Pacific stock market return and volatility in the uncertain world: Evidence from the nonlinear autoregressive distributed lag approach

**DOI:** 10.1371/journal.pone.0285279

**Published:** 2023-05-05

**Authors:** Minh Phuoc-Bao Tran, Duc Hong Vo

**Affiliations:** Research Centre in Business, Economics, and Resources, Ho Chi Minh City Open University, Ho Chi Minh City, Vietnam; Northeast Normal University, CHINA

## Abstract

This paper examines the effects of three distinct groups of uncertainties on market return and volatility in the Asia-Pacific countries, including (i) the country-specific and US geopolitical risks; (ii) the US economic policy uncertainty; and (iii) the US stock market volatility (using the VIX and SKEW indices). Our sample includes 11 Asia-Pacific countries for the 1985–2022 period. We employ the nonlinear autoregressive distributed lag approach (ARDL) estimation technique to capture the asymmetric effects of uncertainties on market return and volatility, which are documented in the literature. Some findings are documented as follows. First, we find that US uncertainty indices, including US geopolitical risk, US economic policy uncertainty, and US VIX, significantly impact Asia-Pacific stock markets, while the impacts of domestic geopolitical risk and the US skewness index (SKEW) are relatively weak. Second, Asia-Pacific stock markets tend to overreact to uncertainty shocks stemming from US economic policy uncertainty and US geopolitical risk. Third, US economic policy uncertainty has more significant effects than the US geopolitical risk. Finally, our research documents that Asia-Pacific stock markets react heterogeneously to good and bad news from US VIX. Specifically, an increase in US VIX (bad news) has a stronger impact than a decrease in US VIX (good news). Policy implications have emerged based on the findings of this study.

## Introduction

Uncertainty demonstrates the lack of understanding of the probabilities of future events that may affect the firm [1]. The effects of uncertainty on the financial markets have been well documented in the literature since the middle of the last century, beginning with Markowitz, Roy, and Tobin [2–4]. These effects have recently continued attracting more attention from scholars, practitioners, and policymakers when the world has experienced the unprecedented COVID-19 pandemic since January 2020 and the Russian evasion of Ukraine since February 2022.

**Fig 1:**
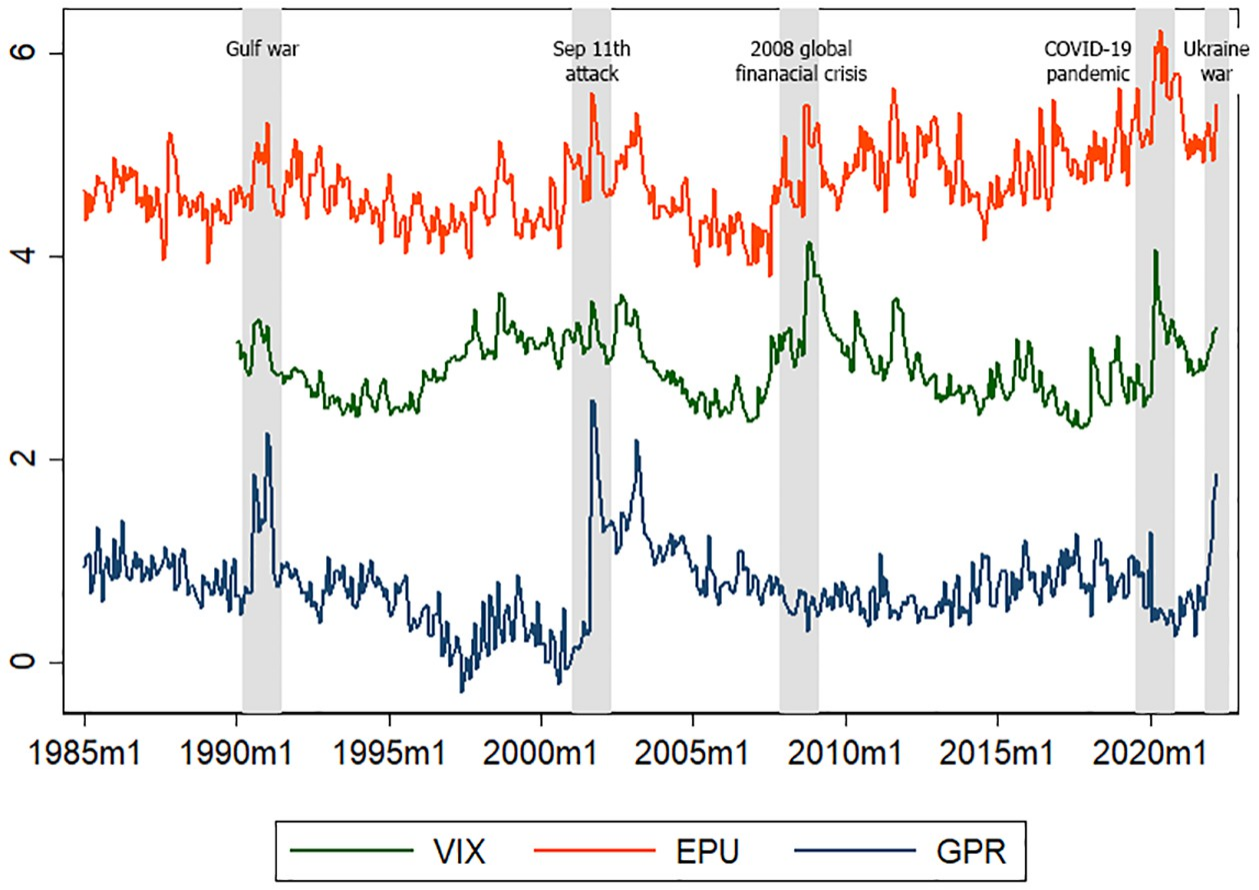
US stock market volatility, geopolitical risk, and economic policy uncertainty. Source: CBOE, policyuncertainty.com; matteoiacoviello.com.

Fig 1 shows the US stock market volatility proxied by CBOE Volatility Index (VIX), US economic policy uncertainty proxied by the economic policy uncertainty index (EPU) and US geopolitical risk proxied by the geopolitical risk index (GPR). The US stock market volatility increases during high economic policy uncertainty and/or high geopolitical risk. The nexus between economic policy uncertainty and stock market volatility has been confirmed by Liu et al. [5], and Baker et al. [6], while the nexus between geopolitical risk and stock market volatility has been confirmed by Smales [7].

Economic policy uncertainty (EPU) denotes the uncertainty deriving from future government economic policies and regulatory frameworks, while geopolitical risk (GPR) represents the uncertainty associated with war, terrorism, and tensions that affect the peaceful environment. These economic-political uncertainties have been documented to impact the economy and the stock markets. The Federal Open Market Committee and the International Monetary Fund (IMF) consider that economic policy uncertainties related to US and European fiscal, regulatory, and monetary policies contributed to the severe economic slump in 2008–09 and the slow recovery in the following years [6]. Meanwhile, 75 per cent of respondents to a Gallup survey in 2017 expressed concern about the economic impact of geopolitical risks posed by military and diplomatic conflicts worldwide. The literature indicates that economic-political uncertainties significantly affect the stock market’s return and volatility. Previous studies consider that increased economic policy uncertainty is associated with increased stock market volatility [6] and decreased market return [8]. Regarding political uncertainties, geopolitical risk is documented to intensify stock market volatility [9] and hurts stock market return [10]. Overall, studies on the effect of domestic political-economic uncertainty on the domestic stock market have been well documented. However, studies on the effect of outside uncertainties, such as the US uncertainties, on domestic stock markets in the Asia-Pacific countries have been underexamined.

The close dependence between the US economy and the Asia-Pacific stock markets has received strong debate in the literature since the global recession [11]. Scholars divide into two opposing schools when it comes to this relationship. The first school holds that the Asian stock markets are less vulnerable to US economic shocks today because their growth is less dependent on the US [12, 13]. Meanwhile, the other school of thought argues that the Asian markets are increasingly dependent on the US stock market and other developed markets due to their nascent size and prominence, thus making them vulnerable to US macroeconomic shocks [14, 15]. Liang et al. [11] is the only study to document relationships between US economic uncertainties on Asian stock markets. Yet, the authors only examine the US economic uncertainties, such as EPU and VIX, while ignoring the political uncertainties (i.e., geopolitical risk) and the US stock market uncertainty (SKEW). Furthermore, Liang et al. [11] focus on the impact of US uncertainties on the stock price rather than their impact on stock market return and volatility.

Given the literature’s divergence and gaps are identified, we attempt to examine how US uncertainties, including US geopolitical risk, US economic policy uncertainty, and US stock market fears (VIX and SKEW indices) and domestic (country-specific) geopolitical risk affect the Asia-Pacific stock markets’ return and volatility. As a result, our study contributes to the extant literature twofold. *First*, this is the first study to examine the effects of the US geopolitical risk and skewness risk on the Asia-Pacific stock markets’ return and volatility. *Second*, we enrich the literature on the effect of US uncertainty on the Asia-Pacific stock markets using a sample covering the unprecedented events globally in the past three years, including the COVID-19 pandemic and Russia—Ukraine conflict. These unprecedented events have received little attention in previous studies. By filling these research gaps, findings from this paper provide useful policy implications for investors and policymakers to manage the adverse effects of the US geopolitical risk and uncertainty shocks on the stock markets’ return and volatility.

### Literature review

In this section, we discuss three important aspects underlying our research framework. The first aspect presents the mechanism by which uncertainty affects stock market return and volatility. Then, we discuss a set of uncertainty variables used in this study, including geopolitical risk (GPR), economic policy uncertainty (EPU), and the US stock market uncertainty (VIX and SKEW indices). And finally, we discuss the asymmetric effects of geopolitical risk, economic policy uncertainty, and the US’s stock market uncertainty on the Asia-Pacific stock markets in the short and long run.

#### Uncertainty and stock markets’ return and volatility

Bernanke [16], Bloom [17], and McDonald and Siegel [18] indicate that economic activity tends to be damaged by uncertainty. On the demand side, businesses are expected to pause their investment needs under the conditions of economic uncertainty [16, 18] while households tighten their spending [19]. On the supply side, uncertainty causes an increase in the cost of hiring labour, which harms the firm’s productivity [17, 20]. Such a negative effect on economic activity may affect business conditions and the stock market [21]. Studies have shown that macroeconomic uncertainty’s effect on stock prices results from adjustments in the required rate of returns [22, 23] or changes in expected future dividends [24]. Such adverse effects can lower business outlook and therefore dampen the stock market performance [6].

#### Uncertainty measures: The geopolitical risk (GPR), economic policy uncertainty (EPU), and the US’s stock market uncertainty (VIX, and SKEW indices)

The uncertainty is documented to exert a profound impact on the stock market. To examine this impact, we adopt the four uncertainty indices covering economic and political dimensions. The first index is the geopolitical risk index (GPR) proposed by Caldara and Iacoviello [25]. GPR measures the risk related to war, terrorism, and tensions among countries that significantly impact international relations and the peaceful environment. The risk is considered to profoundly affect the financial market in general and the stock market since it is often cited as an important factor affecting investment decisions by central bankers, the financial press, and business investors [25]. In terms of economic uncertainty indices, the US economic policy uncertainty index (US EPU), the US stock market’s volatility index (VIX), and the US stock market’s fears about the tail risk index (SKEW) are used. The EPU captures the risk caused by undefined government economic policies and regulatory frameworks in the future. In other words, the EPU index represents the investor’s expectations of future government economic policies. A surge in EPU implies the decline of the market’s confidence in the government’s assertiveness when facing economic threats [15] and/or uncertainty about the impact of future government policies on the business environment [26]. These effects, in turn, severely affect firms’ future cash flow and, therefore, stock price following the discounted cash flow model.

Besides the EPU, the uncertainty indices that also receive the attention of the global stock market are the VIX and SKEW indices. These indices capture the market’s expectations about S&P 500 stock market volatility in the next 30 days. Both indices are considered indicators of fear in the US stock market. Therefore, an increase in VIX and SKEW implies increased fear of the downside risk in the market. However, there is a significant difference between these two indicators. While VIX reflects the fear of risk that comes from frequent events, the SKEW index demonstrates the risk that rarely happens and exerts a profound impact, commonly known as the black swan events. The extant literature indicates that US VIX and SKEW tend to affect the global stock market under the mechanism of trade linkages [27, 28]. However, studies on its impact on the Asia-Pacific market are still scarce. Therefore, in this study, we enrich the literature by employing VIX and SKEW to investigate how these fears affect the return and volatility of the Asia-Pacific stock markets.

#### The asymmetric effects of geopolitical risk, economic policy uncertainty, and the US’s stock market uncertainty on the Asia-Pacific stock markets in the short and long run

Economic variables often have asymmetric properties and other forms of non-linearity. For example, Choi et al. [29] have shown that an increase in oil prices (positive change) has a stronger effect on inflation than a decrease (negative change). In the same vein, Cover et al. [30] reveals that output only responds to a reduction in the money supply shock while not responding to an increase in the money supply. The asymmetric impact of bad and good news on human behaviour has been documented in psychological and economic literature. For example, psychologists indicate that people tend to be attracted to unfavourable information rather than beneficial information [31, 32]. While in economics, loss aversion experiments suggest the same story where people tend to respond more to the utility’s loss (i.e., bad news) than to the gain of equal magnitude (i.e., good news). This statement is widely known as the prospect theory proposed by Kahneman and Tversky [33]. For the explanation, Soroka and McAdams [34] base on the view of evolutionary biology to argue that prioritizing negative information is evolutionary beneficial because the potential costs stemming from adverse information outweigh the potential benefits from negative information. On this basis, we argue that stock market investors react more to bad news than good news stemming from uncertainty. To this end, we employ a nonlinear autoregressive distributed lag approach (NARDL) proposed by Shin et al. [35] for the following rationales. First, it allows us to simultaneously consider both short-term and long-term nonlinear effects of uncertainties. Second, the different effects of positive and negative changes in uncertainty are also well captured using NARDL. Third, NARDL adapts to many data series of different integration orders, including I (0) and I (1). This is suitable for our dataset because of the presence of I (0) and I (1) processes, as indicated by Table 3 below.

Our literature review indicates that the effects of geopolitical risk and economic policy uncertainty on stock market return and volatility are widely investigated. However, the effects of these risks and uncertainties, including the US’s stock market uncertainties, on the Asia-Pacific stock market return and volatility using the nonlinear ARDL, particularly including two major economic and political events such as the COVID-19 pandemic and the Russian invasion of Ukraine, have largely been under-examined. The current lack of empirical evidence on these important risks and uncertainties warrants our analysis to be conducted.

### Data and research methodology

We use monthly data from 11 Asia-Pacific stock market indices to estimate the market return and volatility. Furthermore, a set of uncertainty variables are also employed, including (i) the geopolitical risk index (GPR) developed by Caldara and Iacoviello [25], (ii) the US’s economic policy uncertainty index (EPU) developed by Baker et al. [6], (iii) the US stock market fear index (VIX), (iv) the US stock market fear index about tail risk (SKEW). For VIX and SKEW indices, monthly averages of daily data are used. Our data covers January 1985 and March 2022. All variables are expressed in natural logarithmic form except for the geopolitical risk index (GPR). Table 1 below describes the variables and their data sources.

**Table 1:** A list of the variables and their respective source of data collection.

Variable	Description	Data source
Stock market indices	S&P/ASX200 (Australia); Shanghai Composite (China); Hang Sheng (Hong Kong); BSE Sensex (India); IDX Composite (Indonesia); Nikkei 225 (Japan); FTSE Bursa Malaysia KLCI (Malaysia); PSEi Composite (The Philippines); KOSPI (South Korea); Taiwan Weighted (Taiwan); SET (Thailand)	Investing.com & Yahoo Finance
Uncertainty measures	Geopolitical risk index (GPR)	matteoiacoviello.com
	Economic policy uncertainty index (EPU)	policyuncertainty.com
	CBOE volatility index (VIX)	CBOE
	CBOE skewness Index (SKEW)	CBOE

In this paper, our market returns of each country at month t, *RET*_*t*_, are calculated as follows:

$${RET}_{t}=\text{ln}\left(\frac{P_{t}}{P_{t-1}}\right)$$ (1)

Where *P*_*t*_ is the price of month *t*. Meanwhile, the stock market’s realized volatility of each country at month t, *VOL*_*t*_, is estimated by taking the deviation from the daily price in one month. Specifically,

$${VOL}_{t}=\text{ln}\left(\sqrt{\frac{1}{n-1}{\sum_{k=1}^{n}(P_{k}-\bar{P_{t}})}^{2}}\right)$$ (2)

where *P*_*k*_ is the daily closing price at month *t*; $\bar{P}$ is the mean of daily closing prices of month t; n denotes the number of trading days in month t.

The traditional unrestricted error-correction form of a linear ARDL model is expressed as follows:

$${\Delta y}_{t}=\alpha +{\theta y}_{t-1}+{\delta x}_{t-1}+\sum_{i=1}^{p-1}\gamma_{i}{\Delta y}_{t-i}\;+\sum_{i=0}^{q-1}\pi_{i}x_{t-i}+\;\varepsilon_{t}$$ (3)

Where *α* is the intercept, *γ*_*i*_ and *π*_*i*_ denote the short-run coefficients while *θ* and *δ* denote the long-run coefficients, and *ε*_*t*_ is the error term.

Meanwhile, nonlinear asymmetric long-run co-integrating regression in the NARDL model takes the following form:

$$y_{t}={\sigma^{+}x}_{t}^{+}+{\sigma^{-}x}_{t}^{-}+u_{t}$$ (4)

Where *σ*^+^ and *σ*^−^ are long-run coefficients, *x*_*t*_ is a vector of regressors and is decomposed into its partial sum processes of positive changes ($x_{i}^{+}$) and its partial sum processes of negative changes ($x_{i}^{-}$). They are defined as follows,

$$x_{t}^{+}=\sum_{i=1}^{t}\Delta x_{i}^{+}=\sum_{i=1}^{t}max({\Delta x}_{i},0)\;\text{and}\;x_{t}^{-}=\sum_{i=1}^{t}\Delta x_{i}^{-}=\sum_{i=1}^{t}min({\Delta x}_{i},0)$$ (5)

Combining Eq (4) with Eq (3), we have the general form of the NARDL model as follows.

$${\Delta y}_{t}=\alpha +{\theta y}_{t-1}+\delta^{+}x_{t-1}^{+}+\delta^{-}x_{t-1}^{-}+\sum_{i=1}^{p-1}\gamma_{i}{\Delta y}_{t-i}+\sum_{i=0}^{q-1}(\pi_{i}^{+}{\Delta x}_{t-i}^{+}+\pi_{i}^{-}{\Delta x}_{t-i}^{-})+\varepsilon_{t}$$ (6)

Where *δ*^+^ = −*θσ*^+^ and *δ*^−^ = −*θσ*^−^ while $\pi_{i}^{+}$ and $\pi_{i}^{-}$ are positive and negative short-run adjustments to changes in the explanatory variable, respectively.

Following Shin et al. [35], the steps to estimate the model in Eq (6) using the NARDL estimation technique are as follows. *First*, unit root tests are employed to ensure that our variables used in the model are not in I (2). *Second*, we estimate Eq (4) using the standard ordinary least squares (OLS) to perform the following tests in steps 4 and 5. *Third***,** a bound test is employed to investigate the long-run asymmetric relationship between the magnitudes of the series $y_{t},\;x_{t}^{+}$, and $x_{t}^{-}$ using F-statistics (F_PSS_) proposed by Pesaran et al. [36]. The null hypothesis of F_PSS_ is that “there is no cointegration”. Specifically,

$$H_{0}:\;\theta =\delta^{+}=\delta^{-}=0\;versus\;H_{1}:\;\theta \neq \delta^{+}\neq \delta^{-}\neq 0$$

*Fourth*, when F_PSS_ is significant, meaning “there is cointegration”, we can examine the long- and short-run asymmetry in the relationship between variables using the Wald test. The null hypothesis for detecting the long-run asymmetry is “there is long-run symmetry”. Specifically,

$$H_{0}:\;\delta^{+}=\delta^{-}\;versus\;H_{1}:\delta^{+}\neq \delta^{-}$$

If the Wald test is significant, we reject the null hypothesis, meaning there is long-run asymmetry. The null hypothesis for detecting the short-run asymmetry is “there is short-run symmetry”. Specifically,

$$H_{0}:\;\sum_{i=0}^{q-1}\pi^{+}=\sum_{i=0}^{q-1}\pi^{-}\;versus\;H_{1}:\sum_{i=0}^{q-1}\pi^{+}\neq \sum_{i=0}^{q-1}\pi^{-}$$

If the Wald test is significant, we reject the null hypothesis, meaning there is short-run asymmetry.

#### Empirical results

Table 2 summarises descriptive statistics for variables used in our analysis. Interestingly, the market return in Japan is at the lowest level (0.002), and its market volatility is at the highest level (5.756) compared to other stock markets in the Asia-Pacific region. The highest mean return is for India (0.013), while the lowest mean volatility is for the Malaysian market (2.753). The US exhibits the highest level and volatility of geopolitical risk compared to the Asia-Pacific countries, with a mean of 2,314 and a standard deviation of 1,263, respectively. A high geopolitical risk (GPR) value implies that mentioning topics involved in the risks of war and terrorism is more frequent compared to the Asia-Pacific countries in our sample.

**Table 2:** Descriptive statistics.

Variables	Obs.	Mean	Std. Dev.	Min	Max
LRET_AUS	446	0.005	0.049	-0.552	0.140
LRET_CN	374	0.009	0.118	-0.373	1.020
LRET_HK	446	0.006	0.074	-0.566	0.265
LRET_IND	228	0.013	0.064	-0.273	0.249
LRET_INDO	382	0.006	0.074	-0.379	0.250
LRET_JP	446	0.002	0.059	-0.272	0.183
LRET_MALAY	237	0.003	0.036	-0.165	0.127
LRET_PLIP	222	0.008	0.054	-0.275	0.139
LRET_KOR	446	0.007	0.075	-0.318	0.411
LRET_TW	227	0.006	0.051	-0.209	0.140
LRET_THAI	446	0.006	0.082	-0.359	0.284
LVOL_AUS	447	3.763	0.701	2.034	6.439
LVOL_CN	297	3.832	0.701	2.297	6.020
LVOL_HK	423	5.606	0.840	3.285	7.540
LVOL_IND	297	5.582	0.863	3.065	8.355
LVOL_INDO	384	3.269	1.063	0.520	6.328
LVOL_JP	447	5.756	0.580	3.979	7.518
LVOL_MALAY	340	2.753	0.592	1.129	4.547
LVOL_PLIP	423	4.049	0.729	1.816	6.696
LVOL_KOR	304	3.317	0.502	2.162	5.253
LVOL_TW	297	4.999	0.539	3.810	6.809
LVOL_THAI	304	2.763	0.574	0.969	4.877
GPR_AUS	447	0.083	0.066	0.000	0.450
GPR_CN	447	0.403	0.261	0.070	2.050
GPR_HK	447	0.045	0.057	0.000	0.480
GPR_IND	447	0.207	0.133	0.040	1.130
GPR_INDO	447	0.044	0.050	0.000	0.510
GPR_JP	447	0.231	0.163	0.050	1.240
GPR_MALAY	447	0.034	0.054	0.000	0.970
GPR_PLIP	447	0.054	0.060	0.000	0.480
GPR_KOR	447	0.245	0.225	0.040	1.820
GPR_TW	447	0.047	0.051	0.000	0.340
GPR_THAI	447	0.040	0.034	0.000	0.300
GPR_US	447	2.314	1.263	0.750	13.230
LEPU	447	4.717	0.392	3.807	6.223
LVIX	387	2.911	0.338	2.315	4.138
LSKEW	387	4.792	0.069	4.672	5.052

Note: "L" stands for natural logarithm, "RET" stands for stock market return, "VOL" denotes stock market volatility, "GPR" stands for geopolitical risk, "EPU" represents US economic policy uncertainty, "VIX" stands for US stock market fear, "SKEW" denotes US stock market fears about tail risk. Countries in the sample are denoted as follows: AUS (Australia), CN (China), HK (Hong Kong), IND (India), INDO (Indonesia), JP (Japan), MALAY (Malaysia), PLIP (the Philippines), KOR (South Korea), TW (Taiwan), and THAI (Thailand).

The augmented Dickey-Fuller (ADF) [37] and Philips-Perron (PP) tests [38] are employed to examine the order of integration among the series. Table 3 shows that I (0) and I (1) are present. There is no I (2). These findings imply that our dataset meets the requirements of applying the NARDL estimation approach.

**Table 3:** Unit root test results.

	Augmented Dickey-Fuller test		Phillips–Perron test	
Variables	Level	1^st^ Diff.	Level	1^st^ Diff.
LRET_AUS	-20.891***		-20.906***	
LRET_CN	-6.001***		-19.911***	
LRET_HK	-7.218***		-20.945***	
LRET_IND	-14.175***		-14.24***	
LRET_INDO	-13.298***		-15.911***	
LRET_JP	-19.877***		-19.909***	
LRET_MALAY	-14.535***		-14.602***	
LRET_PLIP	-14.667***		-14.737***	
LRET_KOR	-19.431***		-19.431***	
LRET_TW	-8.819***		-13.535***	
LRET_THAI	-19.107***		-19.085***	
LVOL_AUS	-5.136***		-16.559***	
LVOL_CN	-3.68**		-11.056***	
LVOL_HK	-3.378*		-14.285***	
LVOL_IND	-3.118	-12.783***	-14.395***	
LVOL_INDO	-4.851***		-14.334***	
LVOL_JP	-4.942***		-15.148***	
LVOL_MALAY	-8.076***		-13.421***	
LVOL_PLIP	-3.724**		-17.248***	
LVOL_KOR	-2.91	-7.256***	-14.523***	
LVOL_TW	-4.457***		-13.181***	
LVOL_THAI	-7.833***		-11.659***	
GPR_AUS	-4.611***		-13.859***	
GPR_CN	-2.093	-9.175***	-10.488***	
GPR_HK	-2.428	-8.064***	-12.412***	
GPR_IND	-6.728***		-12.079***	
GPR_INDO	-2.128	-9.489***	-14.355***	
GPR_JP	-6.417***		-11.525***	
GPR_MALAY	-7.610***		-16.734***	
GPR_PLIP	-3.173*		-15.733***	
GPR_KOR	-5.039***		-9.751***	
GPR_TW	-0.095	-9.812***	-9.730***	
GPR_THAI	-10.952***		-17.228***	
GPR_US	-5.148***		-7.915***	
LEPU	-3.692**		-8.493***	
LVIX	-3.49**		-4.463***	
LSKEW	-4.04***		-6.598***	

Note:

*, ** and *** denote the significance level of 10 per cent, 5 per cent and 1 per cent, respectively. The optimal lag orders are selected according to the Akaike information criteria.

Table 4 presents the results of bound tests used to check for nonlinear long-run relationships (cointegration) between variables. These results indicate that Asia-Pacific countries’ market return and volatility show nonlinear long-run relationships with the uncertainty measures, including the geopolitical risk (GPR), the US’s economic policy uncertainty (US EPU), and the US’s stock market uncertainties (VIX and SKEW indices). However, no nonlinear long-run relationships are present between China’s stock market volatility and four uncertainty indices, including GPR, US GPR, VIX, and SKEW indices.

**Table 4:** Results of the bound tests.

	Australia	China	Hong Kong	India	Indonesia	Japan	Malaysia	Philippines	S. Korea	Taiwan	Thailand
Return and GPR	***	***	***	***	***	***	***	***	***	***	***
Return and US GPR	***	***	***	***	***	***	***	***	***	***	***
Return and EPU	***	***	***	***	***	***	***	***	***	***	***
Return and VIX	***	***	***	***	***	***	***	***	***	***	***
Return and SKEW	***	***	***	***	***	***	***	***	***	***	***
Volatility and GPR	***		**	***	***	***	***	***	***	**	***
Volatility and US GPR	***		***	***	***	***	***	***	***	***	***
Volatility and EPU	***	**	***	***	***	***	***	***	***	**	***
Volatility and VIX	***		**	**	***	***	***	***	***	***	***
Volatility and SKEW	***		***	**	***	***	***	***	**	**	***

Note: Only statistically significant values are shown in the table above. Critical values for the FPSS test with unrestricted intercept and no trend are tabulated by Pesaran et al. [36]. The lower and upper bounds with k = 1 are 6.84 and 7.84 at a 1 per cent significance level. The lower and upper bounds with k = 1 are 4.94 and 5.73 at a 5 per cent significance level. The lower and upper bounds with k = 1 are 4.04 and 4.78 at a 10 per cent significance level.

***, ** and * represent the 1 per cent, 5 per cent and 10 per cent significance levels, respectively, meaning | FPSS | exceeds the relevant upper bound.

Table 5 presents the results of the Wald tests used for testing the asymmetry between the increasing effect and the decreasing effect of uncertainty on stock market returns and volatility in both the short and long run. Overall, the results show a long-run asymmetric relationship between SKEW and the stock market returns of India, Indonesia, Malaysia, and the Philippines and; a short-run asymmetric relationship between VIX and returns in Australia, India, Malaysia, Philippines, and Taiwan markets.

**Table 5:** Empirical results of Wald tests.

	Australia		China		Hong Kong		India		Indonesia		Japan		Malaysia		Philippines		S. Korea		Taiwan		Thailand	
	W_LR_	W_SR_	W_LR_	W_SR_	W_LR_	W_SR_	W_LR_	W_SR_	W_LR_	W_SR_	W_LR_	W_SR_	W_LR_	W_SR_	W_LR_	W_SR_	W_LR_	W_SR_	W_LR_	W_SR_	W_LR_	W_SR_
Return and GPR		n/a		n/a	*					n/a				n/a		n/a		n/a		n/a		n/a
Return and US GPR		**		n/a		n/a		n/a		n/a		**		n/a		***		n/a		n/a		n/a
Return and EPU		n/a		n/a		***		n/a								n/a				n/a		
Return and VIX		*		n/a				**						**		***				***		
Return and SKEW				n/a		n/a	*	n/a	**	n/a			*	n/a	***	**		n/a		n/a		n/a
Volatility and GPR	***	n/a		n/a	***	n/a	***	n/a	***	n/a		n/a		***	***		**	n/a		n/a	**	n/a
Volatility and US GPR	***	n/a		n/a	***	n/a	***	n/a	***	***		n/a		n/a	***	n/a	**	n/a		***	***	***
Volatility and EPU	***	***	*	n/a	***	n/a	***	n/a	***	*	**			**	***			n/a		n/a	***	
Volatility and VIX	***			n/a	**		***	n/a	***		**	**		**	***		***	*		n/a	***	*
Volatility and SKEW	***	n/a		n/a	***	n/a	***	n/a	***	n/a		***		n/a	***	n/a		***			***	**

Note: W_LR_ and W_SR_ indicate the Wald statistics for the null hypotheses of long-run and short-run asymmetry. Grey cells represent values that are not statistically significant in Table 5 (bound tests) and, therefore, are not considered in this table.

***, ** and * represent the 1 per cent, 5 per cent and 10 per cent significance levels, respectively, while blank cells represent no statistical significance. A general-to-specific approach is applied. Accordingly, the preferred specification is chosen by starting with max p = 4 and q = 4. After that, all insignificant stationary regressors are removed because inclusion could lead to noise and inaccurate results [39]. “n/a” indicates that the given regressor is eliminated during estimation.

Regarding the stock market’s volatility, we find a long-run asymmetric relationship between uncertainties and stock market volatility in most markets. Besides, a short-run asymmetric relationship between EPU and volatility is also found in Australia, Indonesia, and Malaysia; between VIX and volatility in the Japanese, Malaysian, Korean, and Thai markets; between SKEW and volatility in the Japanese, Korean and Thai stock markets.

#### The effects of domestic and US geopolitical risk on Asia-Pacific stock market return and volatility

Panel A1 of Table 6 shows that increasing country-specific (domestic) geopolitical risk reduces stock market return in Australia, Hong Kong and Japan in the short run. This finding is in line with Yang and Yang [10] when examining the influence of GPR on the US stock market return for the 2000–2019 period. Hoque and Zaidi [40] also find that the country-specific GPR negatively affects the stock market returns in Brazil, Indonesia, South Africa, and Turkey from January 2003 to March 2017. The effect of GPR on the stock market return can be explained in several ways. First, as uncertainty, the GPR can delay consumption and investment decisions [16, 19]. Second, firms are vulnerable to higher costs since GPR curbs global trade and investment and turns globalization into regionalization [20]. Such adverse effects may affect firms’ future cash flows and dampen stock performance.

**Table 6:** NARDL estimation results for the effect of domestic (country-specific) GPR and US GPR on stock market returns and volatility.

	Australia	China	Hong Kong	India	Indonesia	Japan	Malaysia	Philippines	S. Korea	Taiwan	Thailand
**Panel A: The Effect of Country-Specific GPR on Stock Market Return and Volatility**											
*Panel A1*. *The Effect of Country-Specific GPR on Stock Market Return*											
ΔGPR^+^_t_	-0.155***		-0.229**			-0.048*					
ΔGPR^-^_t_				0.298***		-0.097**					
L_GPR_^+^											
L_GPR_^-^											
*Panel A2*. *The Effect of Country-Specific GPR on Stock Market Volatility*											
ΔGPR^+^_t_	1.015*		1.217*	0.483*			3.338**	3.289***			2.890***
ΔGPR^-^_t_						0.494*	3.816**	3.068**			
L_GPR_^+^			-7.674**		-6.473***		-13.229***				
L_GPR_^-^			-8.109**		-6.920***		-13.192***		-0.448*		
**Panel B: The Effect of US GPR on Stock Market Return and Volatility**											
*Panel B1*. *The Effect of US GPR on Stock Market Return*											
ΔUSGPR^+^_t_			-0.010**		-0.013***	-0.012***			-0.015***		-0.017***
ΔUSGPR^-^_t_	0.013**			0.025*		0.032***		0.052***			
L_USGPR_^+^	0.005*					0.008*		0.017*	0.012***		0.021***
L_USGPR_^-^	0.005*					0.008*		0.017*	0.012**		0.021***
*Panel B2*. *The Effect of US GPR on Stock Market Volatility*											
ΔUSGPR^+^_t_	0.103***			0.088**	0.071*	0.083**	0.063*	0.068*	0.064*	0.092***	0.096**
ΔUSGPR^-^_t_					-0.294***						-0.249***
L_USGPR_^+^	-0.177**		-0.388***	-0.367***	-0.394***	-0.196**	-0.308***	-0.378***	-0.241***	-0.328***	-0.228***
L_USGPR_^-^	-0.193**		-0.407***	-0.405***	-0.430***	-0.193*	-0.306***	-0.392***	-0.249***	-0.329***	-0.237***

Note: Only statistically significant values are shown in the table above. L_GPR_^+^ / L_USGPR_^+^ and L_GPR_^-^ / L_USGPR_^-^ indicate the positive and negative long-run coefficients, while ΔGPR^+^ / ΔUSGPR^+^ and ΔGPR^-^/ ΔUSGPR^-^ represent positive and negative short-run coefficients. The optimal lag orders are selected according to the Akaike information criteria.

***, **, and * indicate the significance level at 1 per cent, 5 per cent, and 10 per cent, respectively. China’s long-run coefficients are greyed out because the bound test result in Table 4 shows no nonlinear long-run relationship.

Panel A2 of Table 6 indicates that rising domestic geopolitical risk increases volatility in most markets in the region, particularly in the long run. Economic agents such as business investors, market participants, and central bank officials often consider country-specific geopolitical risks. These agents are important in determining investment decisions and stock market dynamics [25]. They find that real economic activity and stock returns may decline as geopolitical risks increase and capital flows away from emerging economies to advanced economies.

In contrast, in the long run, an increase in US geopolitical risk is found to be associated with an increase in Asia-Pacific’s stock market return. Our finding also aligns with Zaremba et al. [41], which documents that an increase in geopolitical risk is associated with enhancing stock returns in 11 emerging Asian stock markets. This finding also receives support from Erdoğan et al. [42], indicating that a surge in GPR strengthens Turkish stock market return in the long run. A possible explanation is that increased geopolitical risk usually causes a market to overreact, causing returns to drop in the short term. However, this behaviour is corrected later, increasing market return in the long run. Indeed, the phenomenon of heuristic salience in psychology shows that people tend to underestimate the risk of low-frequency events and overreact to the risk of high-frequency events. However, this high frequency can be "artificial high frequencies", which are frequencies that do not occur naturally but are caused by humans, namely the frequency with which information appears on the media [43, 44]. On the other hand, GPR is an index captured by the frequency of information about war and terrorism in newspapers. As such, a high GPR index implies the frequency of news about war and terrorism occurs with high frequency. Such a high frequency of news about war and terrorism, in turn, causes investors to overestimate the risk and thus overreact by selling off. Consequently, the returns fall below their true value. Yet, the investors then realize this and change their behavior, leading to increased stock price and stock return. This overreacting behavior also explains that GPR intensifies volatility in the short term. However, the volatility tends to decrease in the long term.

In summary, Table 6 shows that the effects of US geopolitical risk on the Asia-Pacific stock market return and volatility are more significant than those of country-specific geopolitical risks. This finding is consistent with Bouras et al. [45] study of 18 emerging market economies. This finding can be explained by the globalized nature of modern financial markets, where global shocks are likely to have a larger effect than domestic shocks [45]. On the other hand, the US is the world’s largest financial market in general and the stock market in particular. Therefore, US shocks, such as geopolitical risks, can negatively affect the Asia-Pacific markets more heavily than domestic geopolitical risks.

#### The effect of US economic policy uncertainty on stock market return and volatility in the Asia-Pacific countries

Table 7 presents the effect of US economic policy uncertainty on the Asia-Pacific stock market return. The findings indicate that an increase in US economic policy uncertainty lowers returns and intensifies volatility for most markets in the short run. These findings are consistent with previous studies. For example, Antonakakis et al. [8] find a negative nexus between EPU and US S&P 500’s returns for a sample ranging from January 1985 to January 2013. Similarly, Baker et al. [6] indicate that a 1 per cent increase in EPU is also associated with an approximate 0.43 per cent increase in firm-level stock price volatility in the US. This is because a higher EPU implies a higher unsettled business environment in the future. Such uncertainties may pause firms’ investment needs [16, 18] and consumers’ spending [19]. Furthermore, the uncertainty also causes the cost of hiring labor to surge, dampening the firm’s productivity [17, 20]. Such negative effects can reduce the business prospects and the stock return. In addition, the higher economic uncertainty also leads to flight-to-safety, the phenomenon describing the situation in which investors allocate their portfolios from high-risk assets such as stocks to safer assets. Such a phenomenon would cause the stock market to fluctuate and increase stock market volatility. The finding is like GPR, as the market tends to overreact to the EPU shock, causing the stock price to deviate from its true value. However, investors later realize and adjust their behavior, causing the stock return to increase and volatility to decrease in the long run. In other words, the phenomena of overreaction are found in the effects of both GPR and EPU on market return and volatility in the Asia-Pacific region.

**Table 7:** NARDL estimation results for the effect of the US EPU on stock market return and volatility.

	Australia	China	Hong Kong	India	Indonesia	Japan	Malaysia	Philippines	S. Korea	Taiwan	Thailand
**Panel A: The Effect of US EPU on Stock Market Returns**											
ΔUSEPU^+^_t_	-0.109***		-0.123***	-0.056**	-0.088***	-0.083***	-0.025*	-0.044**	-0.053**	-0.065***	-0.107***
ΔUSEPU^+^_t-1_			-0.044**	-0.055**	-0.052**	-0.034*	-0.028**	-0.036*			
ΔUSEPU^-^_t_											
L_USEPU_^+^		-0.039**							0.029**		0.028**
L_USEPU_^-^		-0.039**							0.029**		0.029**
**Panel B: The Effect of US EPU on Stock Market Volatility**											
ΔUSEPU^+^_t_	0.855***	0.415**	0.646***	0.723***	0.976***	0.651***	0.431**	0.590***	0.429***	0.499***	0.333**
ΔUSEPU^-^_t_					-0.450*		0.431*				
L_USEPU_^+^	-0.411**	-0.908*	-0.938**		-0.461*						
L_USEPU_^-^	-0.451**	-0.941*	-0.992**	-0.535*	-0.541*						

Note: Only statistically significant values are shown in the table above. L_USEPU_^+^ and L_USEPU_^-^ indicate the positive and negative long-run coefficients, while ΔUSEPU^+^ and ΔUSEPU^-^ represent positive and negative short-run coefficients. The optimal lag orders are selected according to the Akaike information criteria.

***, **, and * indicate the significance level at 1 per cent, 5 per cent, and 10 per cent, respectively.

Furthermore, we also find that the effect of the US economic policy uncertainty (US EPU) on the Asia- Pacific stock markets is stronger than the geopolitical risk (GPR). This finding aligns with Hedström et al. [46] when examining ten emerging stock markets from 1995 to 2016. In addition, Das et al. [47] discuss the impact of the US GPR, the US EPU, and US financial stress on emerging stock markets for 1997–2018. The results also show that the effect stemming from EPU is the most dominant effect among these three shocks. This can be explained by the signal precision theory by Pastor and Veronesi [48]. This theory states that volatility in the market is an increasing function of the product of political uncertainty and signal precision. Signals of uncertainty induced by government policies come from an established formal institution. Meanwhile, signals that emerge from rare events such as terrorist attacks or wars arrive at the market randomly, which may not be properly perceivable to investors [49]. As such, the markets react more significantly to the EPU, but they do not react much to the GPR.

#### The effect of US stock market fears on market return and volatility in the Asia-Pacific countries

Table 8 shows empirical results on the effect of the US stock market fear (the VIX index) on market return and volatility in the Asia-Pacific region. Increased fear in the US stock market reduces Asia-Pacific market return. This finding is supported by Sarwar and Khan [50] when examining the effect of US VIX on the returns of emerging markets. Their empirical results confirm that the VIX lowers the stock market return and elevates the stock market volatility from 1^st^ June 2003 to 30^th^ December 2014. The extant literature shows that financial market integration is due to profound trade linkages between countries [27, 51]. In recent decades, Asia has emerged as one of America’s major trading partners, accompanied by the development of financial market integration. On the other hand, the VIX index also reflects the influence of macroeconomic shocks on the US economy [52, 53]. Therefore, a high VIX implies a gloomy outlook for the US economy and threatens the prospects of Asian economies through the trade channel. In other words, an increased VIX could trigger pessimistic sentiment and panic selling in Asia-Pacific markets, dragging down returns and increasing volatility.

**Table 8:** NARDL estimation results for the effect of the US VIX on stock market return and volatility.

	Australia	China	Hong Kong	India	Indonesia	Japan	Malaysia	Philippines	S. Korea	Taiwan	Thailand
**Panel A: The Effect of US VIX on Stock Market Returns**											
ΔVIX^+^_t_	-0.157***	-0.173***	-0.175***	-0.226***	-0.229***	-0.210***	-0.112***	-0.172***	-0.179***	-0.172***	-0.209***
ΔVIX^-^_t_	-0.147***		-0.183***	-0.084*	-0.127***	-0.168***			-0.174***	-0.111***	-0.168***
L_VIX_^+^	-0.011*		-0.019**			-0.025***					
L_VIX_^-^	-0.011*		-0.019*			-0.026***					
**Panel B: The Effect of US VIX on Stock Market Volatility**											
ΔVIX^+^_t_	1.515***	0.921***	1.095***	1.035***	1.347***	1.484***	1.339***	1.041***	1.585***	1.665***	1.017***
ΔVIX^-^_t_	0.601**		0.620**			0.671**	0.639*				0.667*
L_VIX_^+^							0.351**	-0.473*	0.520***	0.566***	
L_VIX_^-^							0.354**	-0.517**	0.494***	0.564***	

Note: Only statistically significant values are shown in the table above. L_VIX_^+^ and L_VIX_^-^ indicate the positive and negative long-run coefficients, while ΔVIX^+^ and ΔVIX^-^ represent positive and negative short-run coefficients. The optimal lag orders are selected according to the Akaike information criteria.

***, **, and * indicate the significance level at 1 per cent, 5 per cent, and 10 per cent, respectively. China’s long-run coefficients are greyed out because the bound test result in Table 4 shows no nonlinear long-run relationship.

Notably, the short-run estimates in Table 8 indicate that the magnitude of the negative coefficients (-) of the VIX on the stock returns are significantly larger than the positive coefficients (+) for Australia, India, and Taiwan. Our Wald test results in Table 5 also confirm the asymmetric effect between positive changes and negative changes in US VIX on the returns of these three markets. This implies that Asia-Pacific stock markets respond more strongly to an increase in VIX (bad news) than to a decrease in VIX (good news). Indeed, psychologists and economists have long documented people’s disproportionate responses to good and bad news. The literature in psychology shows that adverse information is easier to attract attention than favorable information [31, 32]. While in economics, the well-known prospect theory proposed by Kahneman and Tversky [33] also confirms that people tend to react more strongly about the loss in their utility (i.e., bad news) than about the gain (i.e., good news) under an uncertainty environment. It is evolutionarily beneficial to prioritize negative information since the potential costs stemming from adverse information outweigh the benefits deriving from advantageous information. Therefore, the human brain tends to focus on negative information rather than positive information [34].

We continue to examine the impact of the US skewness index (SKEW) on the Asia-Pacific stock markets. Like the VIX, SKEW also reflects the level of fear in the US stock market. However, SKEW differs from VIX in that it reflects fear of events that rarely happen. Empirical results are presented in Table 9. The findings indicate that the impact of SKEW on the Asia-Pacific stock market is quite weak compared to the impact of VIX. This observation reflects the stark difference between both these indices as VIX reflects risk stemming from high probability events while SKEW reflects risk induced by low probability events. In addition, we also find that an increase in US SKEW tends to increase returns and reduce volatility in Asia-Pacific stock markets. This finding aligns with evidence documented by Elyasiani et al. [54]. These authors argue that SKEW is perceived as market greed (fear of missing out on profitable probabilities) rather than market fear (fear of losing money), like VIX.

**Table 9:** NARDL estimation results for the effects of the US SKEW on stock market returns and volatility.

	Australia	China	Hong Kong	India	Indonesia	Japan	Malaysia	Philippines	S. Korea	Taiwan	Thailand
**Panel A: The Effect of US SKEW on Stock Market Returns**											
ΔSKEW^+^_t_	0.300**							0.498**			
ΔSKEW^-^_t_						0.360**			0.482**	0.345**	
ΔSKEW^-^_t-1_	-0.244**			-0.401**		-0.340**	-0.275**		-0.383*	-0.404***	
L_SKEW_^+^								0.230***			
L_SKEW_^-^								0.255***			
**Panel B: The Effect of US SKEW on Stock Market Volatility**											
ΔSKEW^+^_t_			-4.585***		-3.411**			-2.859*		-2.887*	
ΔSKEW^-^_t_	-5.616***								-3.637***	-2.407*	
L_SKEW_^+^											
L_SKEW_^-^											

Note: Only statistically significant values are shown in the table above. L_SKEW_^+^ and L_SKEW_^-^ indicate the positive and negative long-run coefficients, while ΔSKEW^+^ and ΔSKEW^-^ represent positive and negative short-run coefficients. The optimal lag orders are selected according to the Akaike information criteria.

***, **, and * indicate the significance level at 1 per cent, 5 per cent, and 10 per cent, respectively. China’s long-run coefficients are greyed out because the bound test result in Table 4 shows no nonlinear long-run relationship.

### Conclusions and policy implications

Geopolitical risks are risks linked to wars, terrorism and tensions between countries that affect the normal course of international relations [25]. Such an increase in geopolitical risk may negatively affect global stock markets, which are increasingly integrated. Moreover, the US is still a superpower globally, and its decisions may affect other countries. On the other hand, the Asia-Pacific countries have increasingly played an important role in international trade in the currently globalized but uncertain world. As such, examining the effects of the geopolitical risk and uncertainties from the US, the largest economy in the world and closely linked to the Asia-Pacific region, will provide important and meaningful policy implications for the region, particularly during the current Russian invasion of Ukraine. This examination has largely been ignored in the existing literature. As such, this study examines the effects of the US geopolitical risk and economic and market uncertainties on the Asia-Pacific market returns and volatility. The NARDL approach is used to capture the asymmetric effects of uncertainties, namely bad news and good news stemming from uncertainties, on market returns and volatility. We use a sample of 11 Asia-Pacific countries for 1985–2022. Three groups of uncertainties are considered, including (i) country-specific and US geopolitical risks (GPR); (ii) US economic policy uncertainty (EPU); and (iii) the US financial market uncertainties (including VIX and SKEW indices).

Key findings from our study are documented as follows. First, our results confirm that US uncertainty indices, including US GPR, US EPU, and US VIX, exert a significant impact on Asia-Pacific stock markets while the effects of domestic geopolitical risk and US SKEW are relatively weak. Second, in contrast to traditional finance, which assumes that people are rational, we support the view of behavioural finance as findings from our study shows that emotions significantly dominate the Asia-Pacific market. Accordingly, we document that investors tend to overreact significantly to uncertain shocks from geopolitical risk (GPR) and economic policy uncertainty (EPU). Both GPR and EPU reduce returns and increase market volatility in the short term in the Asia-Pacific countries. However, the markets are corrected afterwards because the long-term estimated coefficients of GPR and EPU are positively (negatively) correlated with stock market return (volatility). Third, the Asia-Pacific markets react significantly to the US’s geopolitical risk (US GPR) but little to their domestic geopolitical risks. Fourth, economic policy uncertainty (EPU) provides a stronger impact on the Asia-Pacific markets than GPR. Finally, our research documents that Asia-Pacific stock markets react heterogeneously to positive and negative changes in the US’s stock market uncertainties (US VIX). More specifically, bad news induced by the increase (positive changes) in the VIX index has a stronger impact than good news stemming from the decrease (the negative changes).

Policy implications have emerged based on the findings from our study. First, given that the Asia-Pacific stock markets tend to be most affected by economic policy uncertainty (EPU) than geopolitical risk (GPR), a stable economic policy is recommended to help the stock markets in the region better cope with the ongoing uncertainty caused by two simultaneous shocks: the COVID-19 pandemic and the Russia-Ukraine war. Second, our study provides evidence that the market is substantially dominated by emotions. As such, policies to reassure investors’ psyche should be prioritized in the early stages of uncertainty shocks to minimize the adverse effects on the stock markets. Third, the effects of US uncertainties on Asia-Pacific stock markets are confirmed. These findings can help investors formulate effective strategies to manage their portfolios.
