# Correction: Asia-Pacific stock market return and volatility in the uncertain world: Evidence from the nonlinear autoregressive distributed lag approach

**DOI:** 10.1371/journal.pone.0294104

**Published:** 2023-11-02

**Authors:** Minh Phuoc-Bao Tran, Duc Hong Vo

There are errors in the Funding section. The correct Funding statement is: The research topic was supported by The Youth Incubator for Science and Technology Programme, managed by Youth Promotion Science and Technology Center—Ho Chi Minh Communist Youth Union and Department of Science and Technology of Ho Chi Minh City, the contract number is " 14/2022/HD-KHCNT-VU " signed on 30th, December, 2022. The funders had no role in study design, data collection and analysis, decision to publish, or preparation of the manuscript.
